# Synthesis, characterization and photocatalytic properties of nanostructured lanthanide doped β-NaYF_4_/TiO_2_ composite films

**DOI:** 10.1038/s41598-022-17256-2

**Published:** 2022-08-12

**Authors:** Fabiana M. Pennisi, Anna L. Pellegrino, Nadia Licciardello, Claudia Mezzalira, Massimo Sgarzi, Adolfo Speghini, Graziella Malandrino, Gianaurelio Cuniberti

**Affiliations:** 1 Dipartimento di Scienze Chimiche, Università di Catania, and INSTM UdR Catania, Viale A. Doria 6, 95125 Catania, Italy; 2 Institute for Materials Science, Max Bergmann Centre of Biomaterials and Dresden Center for Nanoanalysis, TU Dresden, 01062 Dresden, Germany; 3 Nanomaterials Research Group, Dipartimento di Biotecnologie, Università di Verona and INSTM, UdR Verona, Strada Le Grazie 15, 37134 Verona, Italy; 4 Present Address: ST Microelectronics, Strada Primosole 50, 95121 Catania, Italy; 5 Present Address: Department of Drug and Health Sciences, University of Catania, Viale Andrea Doria 6, 95125 Catania, Italy; 6 Present Address: Department of Molecular Sciences and Nanosystems, Ca’ Foscari University of Venice, Via Torino 155, 30172 Venezia Mestre, Italy

**Keywords:** Chemistry, Materials science

## Abstract

The photocatalytic approach is known to be one of the most promising advanced oxidation processes for the tertiary treatment of polluted water. In this paper, β-NaYF_4_/TiO_2_ composite films have been synthetized through a novel sol–gel/spin-coating approach using a mixture of β-diketonate complexes of Na and Y, and Yb^3+^, Tm^3+^, Gd^3+^, Eu^3+^ as doping ions, together with the TiO_2_ P25 nanoparticles. The herein pioneering approach represents an easy, straightforward and industrially appealing method for the fabrication of doped β-NaYF_4_/TiO_2_ composites. The effect of the doped β-NaYF_4_ phase on the photocatalytic activity of TiO_2_ for the degradation of methylene blue (MB) has been deeply investigated. In particular, the upconverting TiO_2_/β-NaYF_4_: 20%Yb, 2% Gd, x% Tm (x = 0.5 and 1%) and the downshifting TiO_2_/β-NaYF_4_: 10% Eu composite films have been tested on MB degradation both under UV and visible light irradiation. An improvement up to 42.4% in the degradation of MB has been observed for the TiO_2_/β-NaYF_4_: 10% Eu system after 240 min of UV irradiation.

## Introduction

The presence of pollutants in water has raised increasing concern because of the adverse consequences for human health and wildlife, arising from their release into the environment^1–4^.Pollutants may be spread into the aquatic environment via several different sources, such as improperly treated industrial by-products, hospital and municipal wastewater, soil/groundwater systems or into the atmosphere through rainwater^5^, and some of them can act as persistent pollutants. In this scenario, innovative strategies to clean wastewater are critically important, given the limited availability of fresh water on Earth and the increasing of global population. Pharmaceuticals, e.g. antibiotics, and dyes are among the most worrying threats to water bodies. Indeed, pharmaceuticals and, primarily, antibiotics represent a considerable threat linked with their potential to increase antibacterial resistance, putting at risk both environmental and human health^6^.On the other hand, the textile industry uses dyes in large amounts, which, mainly in developing countries, are released into the effluents creating a menace to aquatic life due to the carcinogenic and toxic effects of dyes^7,8^. The removal of organic dyes from the environment has been a problem over the years for many industries and the society as a whole. There has been a great effort to degrade these substances reducing their impact on the environment using various techniques, including e.g. adsorption, biodegradation and advanced oxidation processes^9–11^. Therefore, it is crucial to boost research in the field of new nanomaterials for wastewater treatment. Advanced oxidation processes (AOPs) and, especially, photocatalysis are of particular interest in this scenario because they are cost-effective, relatively easy-to-use and allow to remove traces of persistent pollutants, reaching, ideally, total mineralization. Since the early discovery of its photocatalytic activity^12^, TiO_2_ has been one of the most used and investigated photocatalysts, due to its excellent catalytic activity, low cost, large surface area and relatively low toxicity^13^. However, especially in the most commonly used crystalline anatase phase, TiO_2_ suffers from the drawback of possessing a large bandgap (~ 3.2 eV) which, in turn, implies the use of UV light irradiation for its activation. Therefore, over the years, several methods, including metal and non-metal doping, decoration with noble metals and combination with binary or ternary selenide nanoparticles, have been investigated to enhance the activity of TiO_2_ under visible-light by reducing its bandgap and/or by decreasing its electron–hole pair recombination rate^14–21^. The need to obtain also a product with a vast range of applications exploiting the largest available energy source, i.e. the sun, has led to the synthesis of particles capable of absorbing low energy photons from the sun and converting these into high energy photons able at degrading various organic substances.

More recently, combining upconverting nanoparticles with TiO_2_ or other wide bandgap semiconductors has been considered another alternative for sunlight-driven photocatalytic processes^22–25^. In some cases, systems based on the incorporation of upconverting nanomaterials in TiO_2_ films and nanostructures have been recently investigated for their photocatalytic properties under near infrared (NIR) light^26–30^. Upconverting nanomaterials, indeed, show the appealing property to be able to emit in the visible and/or UV regions when irradiated with NIR or visible light. Therefore, coupling them with TiO_2_ would offer the possibility to better exploit lower energy ranges for the activation of the photocatalyst.

However, in the very few reported studies on this topic, often the photolysis of the studied pollutant under the same conditions used for the photocatalytic reaction is missing. Another crucial issue in heterogeneous photocatalysis is the form of the catalysts, which can be used as a slurry (nanoparticle dispersion)^31^ or immobilized on a support in form of film or membrane^32^. If on the one hand, slurry photocatalysts have the advantage of a larger available surface area for the adsorption and consecutive faster photo-degradation of the pollutants, on the other hand, their recovery and recycling are pretty challenging. The application of TiO_2_ in real wastewater treatment conditions is, indeed, still limited by the risks associated with TiO_2_ release into the environment. There is a need for engineered systems, which allow for an extensive surface coverage with TiO_2_, and an easy recovery and recycling, linked with a limited or negligible loss of photocatalyst during the process. In this direction, immobilized photocatalysts can play a crucial role^32,33^.

Herein, we report a full engineering of β-NaYF_4_/TiO_2_ nanostructured composite films, which have been obtained, for the first time, through an innovative sol–gel and spin-coating procedure, with the fluoride component appropriately doped with lanthanide ions to induce upconversion (UC) or downshifting (DS) properties. A mixture of β-diketonate complexes of Na and Y (Na(hfa) ·tetraglyme and Y(hfa)_3_·diglyme) has been successfully employed together with Yb^3+^, Tm^3+^, Gd^3+^ and Eu^3+^ analogous adducts (Ln(hfa)_3_·diglyme) as doping sources for the production of the doped β-NaYF_4_ component. In particular, through a fine tuning of the precursor mixtures, the upconverting TiO_2_/β-NaYF_4_: 20% Yb, 2% Gd, x% Tm (x = 0.5 and 1%) and the downshifting TiO_2_/β-NaYF_4_: 10% Eu composite films have been successfully synthesized and deeply characterized to assess their structural, morphological, compositional and luminescence properties. Finally, for the fabrication of a heterogeneous photocatalyst devices, the energy conversion component mixed with the titanium (IV) oxide nanoparticles has been immobilized on glass substrates. The study of the photocatalytic activities of the doped β-NaYF_4_/TiO_2_ composite films has been performed for the degradation of organic dyes such as methylene blue (MB) under both visible or UV light irradiation.

## Results and discussion

### Synthesis and characterization of the β-NaYF_4_/TiO_2_ nanocomposites

**Figure 1:**
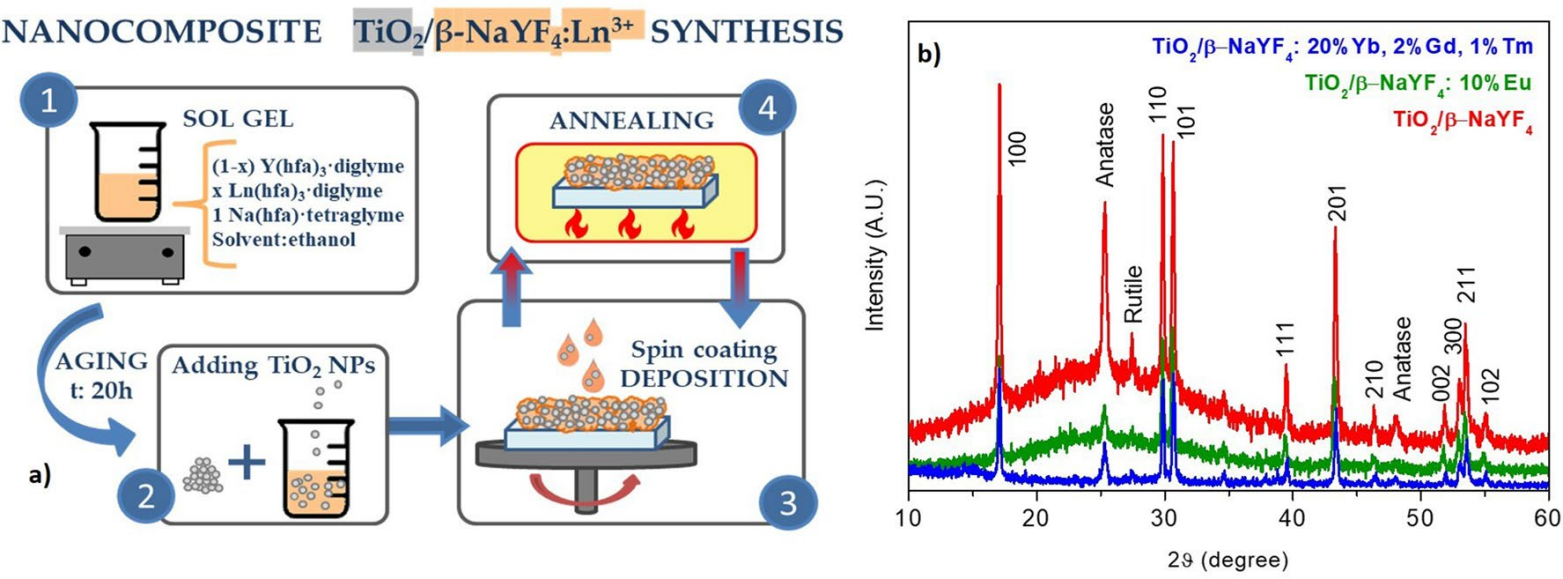
(**a**) Scheme of the composite synthesis from the sol–gel formation to the film deposition. (**b**) XRD patterns of undoped TiO_2_/β-NaYF_4_ composite and doped with different amounts of doping ions Yb^3+^, Gd^3+^ and Tm^3+^, and Eu^3+^ [deposition temperature: 400 °C; substrate: glass for sample in red and green, and Si for sample in blue].

The starting point of the novel composite fabrication process is a straightforward and simple sol–gel approach to produce a pure and nanostructured β-NaYF_4_ component. The composite has been synthesized by suspending TiO_2_ P25 nanoparticles in a gel of Na-Y-F obtained after 20 h aging, using a mixture of β-diketonate complexes of Na(hfa)·tetraglyme, Y(hfa)_3_·diglyme and the related doping elements for the upconversion (Yb^3+^, Gd^3+^, Tm^3+^) or downshifting (Eu^3+^) properties (Fig. 1a). The β-diketonate complexes used as precursors for the sol–gel approach represent a single source of metal elements and fluorine for the β-NaYF_4_ phase formation. The Yb^3+^, Gd^3+^, and Tm^3+^ dopants have been chosen because it has been reported that this type of systems, upon excitation in the NIR, displays emission properties not only in the visible region, but also in the UV range, which is useful for the anatase activation^34^. In parallel, it has been recently reported that the Eu^3+^ and Tb^3+^ doping enhances the TiO_2_ photocatalytic activity^35^. The present approach allows an easy and fine-tuning of the doping ion composition by just changing the starting mixture ratios and thus tailoring the luminescence properties of the final composite material.

The obtained mixture has been spin-coated on glass and Si substrates, following a procedure previously applied for the formation of β-NaYF_4_ films^36^. An in-depth study has been carried out by varying the dopant nature and their respective percentages to establish the reproducibility of the present approach and to evaluate the most promising systems in terms of homogeneity and photocatalytic performance. Among them, the systems reported in Table 1 will be discussed in detail, both in terms of characterization and photocatalytic performances.

**Table 1:** List of samples used to photo-degrade an aqueous solution 1.8 × 10^–5^ M MB under visible and UV light irradiation.

Composite sample supported on glass	Sample	Composite layer property
Pure TiO_2_/β-NaYF_4_	1-blank	Blank sample
TiO_2_/β-NaYF_4_: 20%Yb, 2%Gd, 1%Tm	2-UC	Upconverting
TiO_2_/β-NaYF_4_: 20%Yb,2%Gd, 0.5%Tm	3-UC	Upconverting
TiO_2_/β-NaYF_4_: 10%Eu	4-DS	Downshifting

For all the samples, the XRD characterization shows the formation of the pure hexagonal β-NaYF_4_ phase, which represents the best host lattice for the doping ion luminescence activities compared to the cubic phase. In Fig. 1b, the patterns of the pure TiO_2_/β-NaYF_4_, and the doped TiO_2_/β-NaYF_4_: 20% Yb, 2% Gd, 1% Tm, and TiO_2_/β-NaYF_4_:10% Eu systems (deposited on glass) show reflections at 2θ = 17.10, 30.06, 30.78, 39.63, 43.49, 53.24, 53.65, 55.20°, associated with those of the hexagonal NaYF_4_ structure, in accordance with the ICDD no. 16–0334. The bump centered around 25° is due to the amorphous nature of the glass substrate. In addition, the peaks at 2θ = 25.32 and 48.07° are related to the 101 and 200 reflections of the TiO_2_ component as anatase structure (ICDD no. 00–084-1286), with a very small peak at 27.34° due to the 110 reflection of the rutile phase (ICDD no. 00–077-0443).

The comparison of the XRD patterns of pure and doped TiO_2_/β-NaYF_4_ composites evidences that the peak positions of the co-doped films correspond perfectly to the peak positions of the undoped phase, suggesting that the doping ions are substitutional to Y ones in the NaYF_4_ lattice. Thus, the Ln^3+^ and Y^3+^ ions are located in the same ninefold coordination environment^37^, and the lattice structure does not present any distortion because of the similar ionic radii of the Yb^3+^ (1.042 Å, ninefold coordination), Tm^3+^(1.052 Å, ninefold coordination), Er^3+^ (1.062 Å, ninefold coordination) and Y^3+^ (1.075 Å, ninefold coordination)^38^.

**Figure 2:**
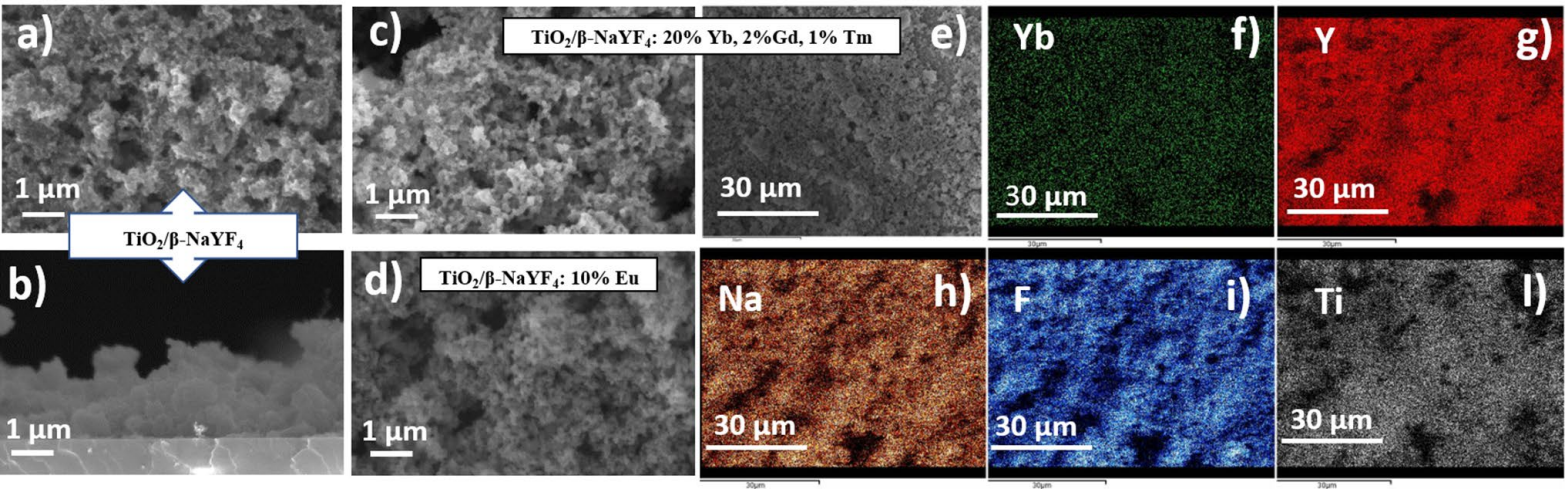
FE-SEM images of: TiO_2_/β-NaYF_4_ deposited at 400 °C on Si (100), (**a**) plan view and (**b**) cross section; TiO_2_/β-NaYF_4_: 10% Eu (**d**). TiO_2_/β-NaYF_4_: 20% Yb, 2% Gd, 1% Tm deposited at 400 °C on Si (100) (**c**) plan view and EDX elementary maps (**e-l**) of Yb, Y, Na, F and Ti elements.

The field-emission scanning electron microscopy (FE-SEM) analysis in Fig. 2 has allowed to study the effects of doping ions on the film morphologies, confirming the homogeneity of the TiO_2_/β-NaYF_4_ composites over large areas (2 × 3 cm). This characterization has been carried out on Si substrates to avoid the charging effect due to the glass substrate. The undoped TiO_2_/β-NaYF_4_ composite film deposited on Si in Fig. 2a displays a very homogeneous and porous structure, which can be described as a sponge-like morphology. The cross-section image in Fig. 2b confirms the formation of a very porous structure with thickness ranging from 1.2 to 1.8 μm. The analogous TiO_2_/β-NaYF_4_: 20% Yb, 2% Gd, 1% Tm (Fig. 2c) and TiO_2_/β-NaYF_4_: 10% Eu films on Si (Fig. 2d) show a similar porous morphology with grains of hundreds of nanometers. Generally, only slight changes of the morphologies are observed in these samples and may be related to the different effects of doping ion concentration. The porous structures, indeed, may be likely associated with the formation of the composite systems with the growth of the NaYF_4_ component around the TiO_2_ nanoparticles during the densification process and annealing treatment. The mesoporous morphologies obtained in the present approach are highly desirable for an optimized engineering of a heterogeneous photocatalyst. Indeed, on the one hand, this structure maximizes the interaction of the two components in the composite and, on the other hand, presents a wider active surface area for the irradiation process.

Additionally, the composition of the doped films has been checked through EDX analysis on different sites and it has been confirmed that it corresponds to the starting mixture ratio of the main components and the doping ion percentage. The EDX spectrum of the TiO_2_/β-NaYF_4_: 20% Yb, 2% Gd, 1% Tm composite (Fig. S1) shows the typical peaks of the Na, Y, and F elements together with the peak observed at 1.6 keV due to Yb Mα for the doped system. In addition, the Ti K lines in the 4.5–5.1 keV range and O Kα at 0.520 keV are observed due to the presence of the TiO_2_ nanoparticles. Gadolinium and thulium peaks, relative to the nominal concentration of 2% and 1%, are scarcely detectable due to their low concentrations that are on the borderline of the detection limit of the EDX technique. However, their presence has been confirmed by their photocatalytic and luminescence properties described below. The absence of a signal at 0.277 keV, relative to C Kα peak, allows to exclude any carbon contamination due to the β-diketonate precursors.

Moreover, the homogeneity of the film is assessed through EDX maps on TiO_2_/β-NaYF_4_: 20% Yb, 2% Gd, 1% Tm nanocomposite (Fig. 2e-l). A large area has been analyzed in order to confirm the homogeneity of the systems, and the colored maps display a uniform distribution of Ti, Na, Y and F elements in the formation of a well distributed nanocomposite. The distribution of Yb doping element confirms the homogeneous doping of the system.

Analogously, the compositional analysis of the TiO_2_/β-NaYF_4_: 10% Eu composite deposited on Si has been assessed via EDX. The spectrum is reported in Fig. S2 and shows the typical peaks of the Na, Y, and F elements. In particular, in addition to the Na Kα and Y L peaks, the L peaks due to the Eu doping ion and Ti and O K lines are visible. Fig. S3 shows elemental mapping images for the TiO_2_/β-NaYF_4_: Eu^3+^ nanocomposite. These elemental mapping images, correlated with the SEM image, show that the elemental distribution is very homogeneous.

### Photocatalytic activity

The photocatalytic activities of the composites have been probed for the degradation of methylene blue (MB), a known model pollutant and standard dye in photocatalytic experiments^39^. In particular, the degradation of MB is a recommended test for photocatalytic activity in the ISO/CD10678 and it is also used in photocatalysis with upconverting systems^22,29,30^. In addition, MB is also commonly used for biological staining as well as coloring paper, hair, cottons and wools, thus it represents a common pollutant in wastewater which needs to be removed^40^.

Specific experiments have been designed to prepare several identical samples, during the same fabrication process, to test the composite photocatalytic activity under visible light or UV light irradiation. The photocatalytic efficiency of the composites has been tested by monitoring the photodegradation of MB through UV–Vis absorption spectroscopy. A concentration of MB of 1.8 × 10^−5^ M has been used, in line with the literature reports on the topic^33,41–43^, where the concentration of the MB ranges from 1.5 × 10^−5^ M^41^ to 3.8 × 10^−5^ M^43^.

In particular, MB shows a maximum absorption peak at about 663 nm, which gradually diminishes upon exposure to visible light, or UV light irradiation in the presence of a photocatalyst, as a consequence of its degradation.

#### Photocatalytic activity of the upconverting β-NaYF_4_/TiO_2_: Yb^3+^, Gd^3+^, Tm^3+^ composite under visible light

The photocatalytic activity of the films doped with lanthanides such as Yb^3+^, Gd^3+^, Tm^3+^ (2-UC and 3-UC samples) with different concentrations, has been assessed by their photocatalytic decomposition of an aqueous MB solution.

In a first experiment, the photolysis of methylene blue has been investigated. The graph (Fig. S4) shows how the MB absorbance values decrease on increasing the exposure time to visible light. Methylene blue intrinsically exhibits a high photolysis, which means it degrades by simple exposure to visible light, and, indeed, it shows a degradation of 33.7% after 240 min (Fig. S4). In a second experiment, the degradation of methylene blue in the presence of the β-NaYF_4_/TiO_2_ blank composite has been investigated. The graph does not show any substantial differences compared to photolysis (Fig. S4). This may be explained considering that TiO_2_ P25 particles have an anatase/rutile ratio of about 80/20. Although the rutile plays an important role in the photocatalytic activity of TiO_2_ P25 particles under visible light^15^, given its low percentage in the whole used samples and the absence of dopants in the composite, the contribution of the weak photocatalytic activity is less significant than the one of the strong photolysis of MB under visible light irradiation.

**Figure 3:**
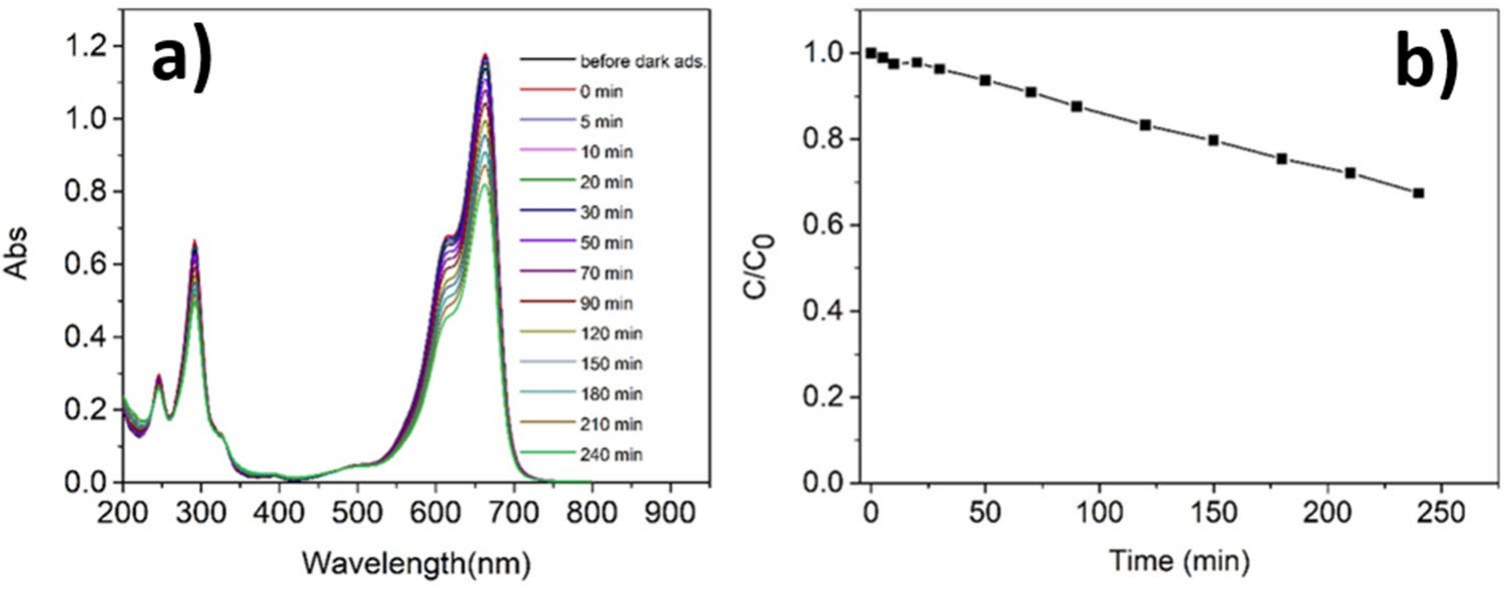
Photo-degradation of an aqueous solution of MB 1.8 × 10^–5^ M in the presence of sample 3-UC, namely TiO_2_/NaYF_4_: Yb^3+^(20%), Gd^3+^(2%), Tm^3+^(0.5%) under visible light irradiation: (**a**) variation of the UV–Vis absorption spectra of the MB solution in time during the photodegradation (the first spectrum represents the spectrum acquired before the 30 min of dark adsorption and the zero time is exactly at 30 min after dark adsorption); (**b**) Variation of the concentration of MB in time during the photodegradation.

Subsequently, the 2-UC and 3-UC samples have been tested to investigate whether the photocatalytic activity increases in the presence of lanthanide dopants. Similar results have been obtained for the two samples, thus data from 3-UC sample are commented in the following and the results obtained with 2-UC sample are reported in the supporting information (Fig. S5). The presence of the lanthanide doping ions, Yb^3+^(20%), Gd^3+^(2%), Tm^3+^(0.5%), inducing upconversion does not affect the photocatalytic properties (Fig. 3a,b), since the MB degradation percentage (32.5%) is once again comparable with the methylene blue photolysis.

Present results indicate that the Ln-doped NaYF_4_ does not play a role in improving the photodegradation of methylene blue under visible light irradiation. Assessed the upconversion properties of the 2-UC and 3-UC composites (vide infra), the negligible effect has to be ascribed to the lamp used in this study, which might not have the right irradiance in terms of the desired wavelengths and power needed for the excitation of upconverting phenomena. These findings may be compared with literature data. An enhancement of the photocatalytic activity has been observed for the upconversion-TiO_2_ systems only when a 980 nm laser has been used, while when photocatalytic measurements are carried out using visible light, no improvement of the photocatalysis has been observed. This issue has been clearly addressed in the ref. ^44^ of Sahu et al., who disproved the role of UC in catalytic enhancements observed for Ln^3+^ phosphor-semiconductor composites under visible light irradiation.

#### Photocatalytic activity of the downshifting TiO_2_/β-NaYF_4_:10% Eu composite under UV light

**Figure 4:**
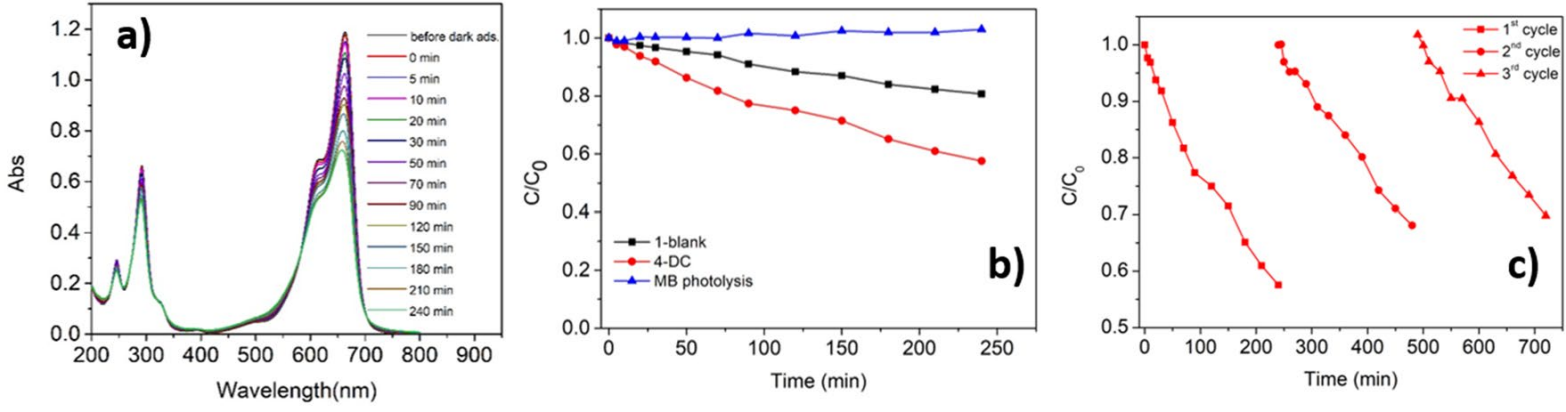
Photodegradation of an aqueous solution of MB 1.8 × 10^–5^ M: (**a**) variation of the UV–Vis absorption spectra of MB under UV irradiation in the presence of the photocatalyst 4-DS; (**b**) variation of the concentration of MB in time through photolysis (blue line), photocatalysis with 1-blank (black line) and 4-DS (red line) samples; (**c**) recycling experiments: variation of the concentration of three solutions of MB (1.8 × 10^–5^ M) versus time under UV irradiation in the presence of the same 4-DS sample reused for three times.

First of all, the photolysis of methylene blue under UV irradiation has been studied. As expected, being the MB a standard dye in the UV region, a negligible degradation is observed under UV irradiation (Fig. S6). The degradation of methylene blue in the presence of the TiO_2_/NaYF_4_ composite has been investigated as well (1-blank, Fig. 4b). The observed large difference between the initial and final maximum absorbance values of the MB solution, as the exposure time increases, indicates a degradation of 19.3%. This degradation behavior is due to the photocatalytic activity of the anatase component (80%) of the TiO_2_ nanoparticles.

The degradation of methylene blue in the presence of the 4-DS sample, i.e. the Eu doped NaYF_4_/TiO_2_ composite, has been successively investigated and is reported in Fig. 4a. The most intense spectrum in the figure represents data acquired before the 30 min of dark adsorption, while the zero time is exactly at 30 min after dark adsorption. This system is more effective and a degradation of 42.4% is observed after 240 min. In this case, an enhancement of 120% with respect to the 1-blank sample (Fig. 4b) is found and can be ascribed to the strong effect of the downshifting component.

This behavior may be interpreted considering the recent results reported for the Eu^3+^ doped TiO_2_ systems. It has been proposed that Eu^3+^ doping successfully enhances the photocatalytic activity of TiO_2_, by reducing the recombination of electron/hole pairs by trapping photogenerated electrons^35,45,46^. In the present case, it seems reasonable that the Eu^3+^ doped NaYF_4_ system plays a similar role, decreasing the electron–hole recombination of the TiO_2_ anatase component under UV irradiation, thus enhancing the photocatalytic activity.

The photocatalytic degradation curves obtained for the samples of 4-DS and of 1-blank have been fitted using a pseudo-first-order kinetic model as in Eq. 1:^47^

1 $$-ln\frac{{C}_{t}}{{C}_{0}}=kt$$

where C_t_ is the concentration of MB at time t, C_0_ is the concentration of MB at time zero and k is the degradation rate constant. The slope of the fitted curve gives the value of the degradation rate constant, k. The linear relationship of − ln(C_t_/C_0_) vs. time for the samples 4-DS and 1-blank is shown in Figs. S7 and S8, respectively. The calculated values for k are reported in Table 2. As can be seen, the degradation rate constant for MB photo-degradation in the presence of the sample 4-DS is about 2.5 times the value of the one observed for the same reaction in the presence of 1-blank. The present results confirm the promising photocatalytic activity of the 4-DS composite, paving the way for a massive application of the present synthetic strategy to produce hetero-catalyst systems.

**Table 2:** Degradation rate constants for MB photo-degradation in the presence of the 4-DS and the 1-blank samples.

Catalyst	k_MB_ (min^-1^)
4-DS	2.26 × 10^–3^
1-blank	8.75 × 10^–4^

Given the promising results obtained for the sample TiO_2_/NaYF_4_:Eu^3+^ in the MB photodegradation under UV irradiation, recycling experiments have been performed. The sample has been recovered after the photocatalytic degradation, washed with water and reused in the photodegradation of a new freshly prepared MB solution. This procedure has been repeated 3 times (Fig. 4c).

The MB degradation is around 42.4% during the first use of the sample 4-DS, then it drops to 32% during the second use of the sample and to 30.2% during the third use. This means that after 3 cycles, there is a drop of only 12.2% in the efficiency of the photo-degradation of MB using the same 4-DS sample. The 4-DS sample, after the 3-recycling tests, has been characterized through XRD and FE-SEM measurements to ascertain if the sample remained intact after the repeated uses. Both analyses show that the structure and morphology of the sample are unchanged after the three photocatalytic cycles (Fig. S9). These results are promising and show the potential of these systems in industrial applications where several reutilizations before sample discard are required.

### Luminescent properties of the nanocomposites

**Figure 5:**
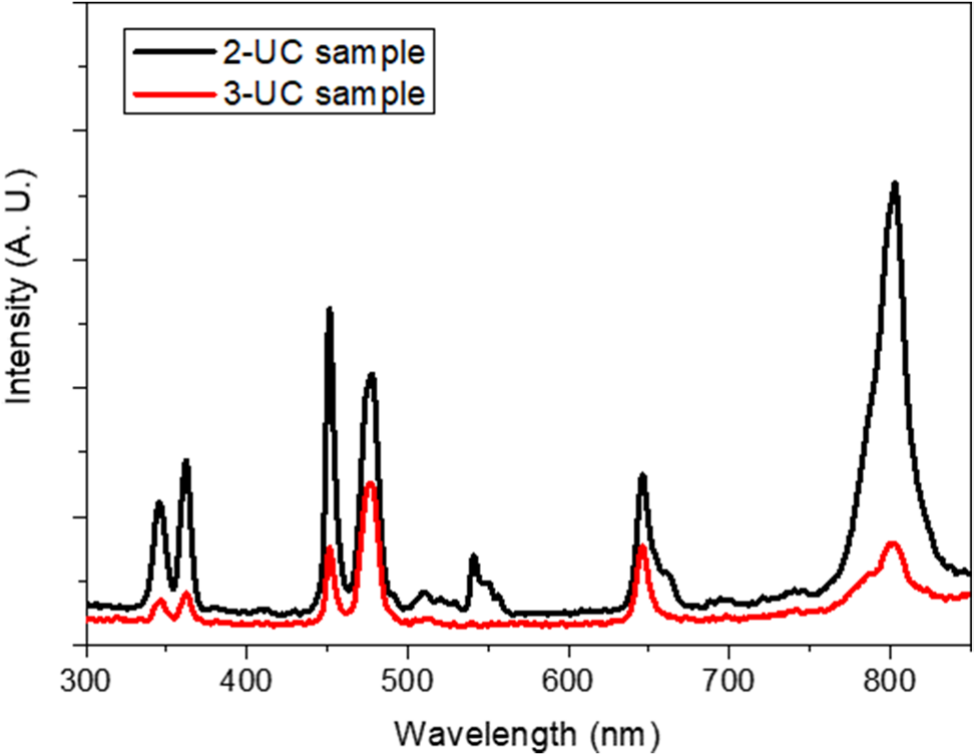
Upconversion spectra for the TiO_2_/β-NaYF_4_: 20% Yb, 2% Gd, 1% Tm (2-UC, black line) and TiO_2_/β-NaYF_4_: 20% Yb, 2% Gd, 0.5% Tm (3-UC, red line) nanocomposites under 980 nm excitation.

Upconversion emission has been measured for the TiO_2_/β-NaYF_4_: 20% Yb, 2% Gd, 1% Tm (2-UC) and TiO_2_/β-NaYF_4_: 20% Yb, 2% Gd, 0.5% Tm (3-UC) nanocomposites. The spectra are shown in Fig. 5, where strong emission bands are assigned to the Tm^3+^ ions, sensitized by the Yb^3+^ ions. In fact, upon excitation at 980 nm, the Yb^3+^ ions are excited and energy transfer processes to the Tm^3+^ ions are active. Then, emissions due to Tm^3+^ transitions are observed, as clearly visible in the spectra shown in Fig. 5, while the very weak emission around 550 nm observed for the 2-UC sample is most probably due to small Er^3+^ ion impurities. Importantly, a quite strong UC band in the UV region (around 350 nm) is observed, pointing to a very good upconversion efficiency of the Ln-doped NaYF_4_ component. Thus, the lower efficacy of the composite in the photocatalytic properties with respect to the other samples, could be due to the relatively low efficiency of the upconversion process when the sample is irradiated by a conventional lamp. These findings are confirmed by the luminescence studies which have been carried out on the samples after their applications in the photo-degradation cycles.

**Figure 6:**
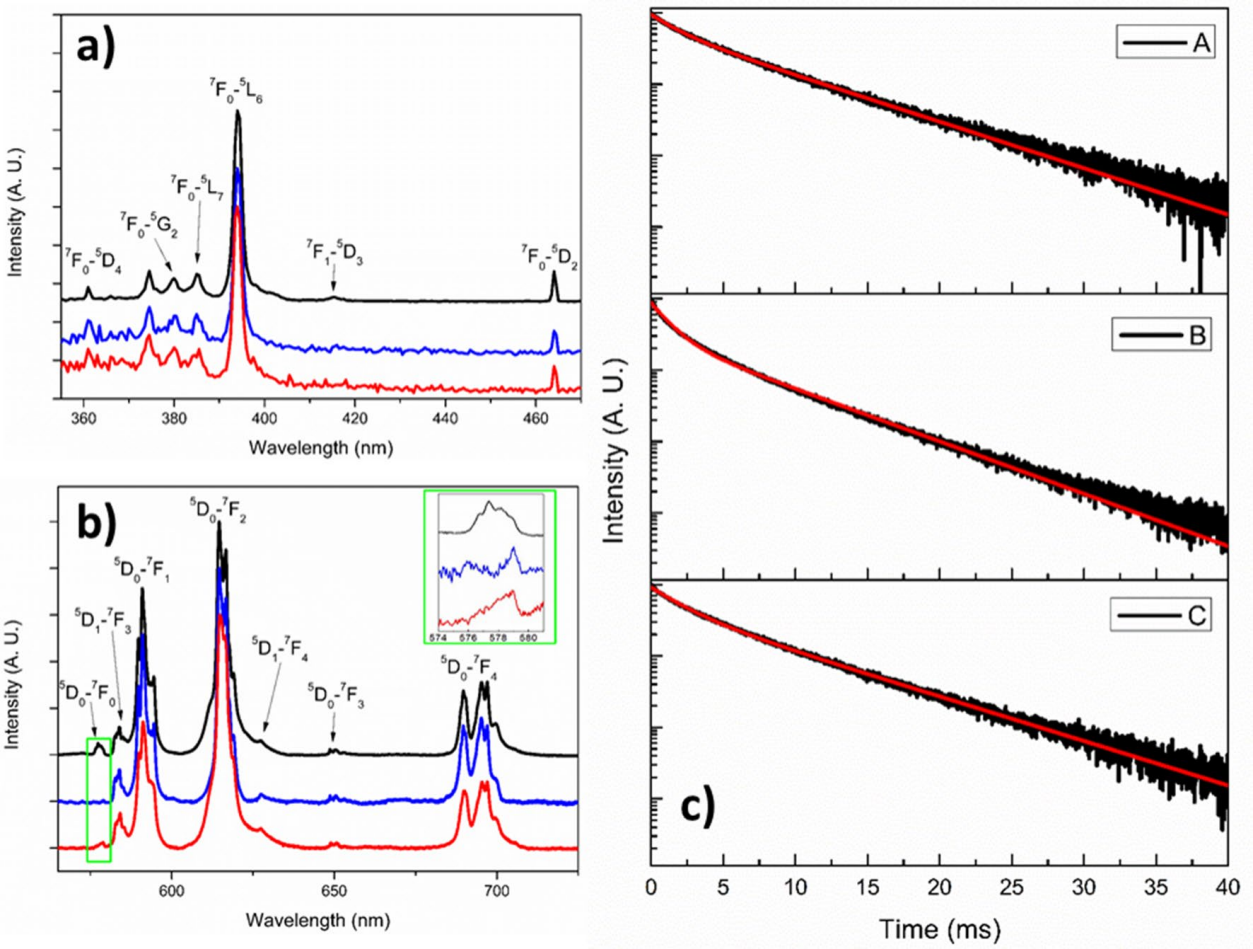
(**a**) Luminescence excitation spectra (λ_em_ = 614 nm) and (**b**) emission spectra (λ_exc_ = 532 nm) for the TiO_2_/NaYF_4_: 10% Eu (4-DS sample) nanocomposites: as-prepared (black line); after one photocatalytic cycle in the presence of methylene blue (blue line); after three photocatalytic cycles in the presence of methylene blue (red line). Emission decay curves of the band at 613 nm (**c**) for the TiO_2_/β-NaYF_4_: 10% Eu^3+^ nanocomposites: as prepared sample (**A**); after one photocatalytic cycle (**B**); after three photocatalytic cycles (**C**), in the presence of methylene blue. The corresponding fits, using Eq. 2, are indicated by red lines.

In Fig. 6a, the excitation spectra registered at the emission wavelength of 614 nm for the TiO_2_/NaYF_4_: 10% Eu nanocomposite samples (as-prepared, after one photocatalytic cycle, and after three photocatalytic cycles in the presence of methylene blue) are shown. The excitation spectra show typical bands assigned to transitions of the Eu^3+^ ions. The most intense band is observed at 394 nm, assigned to ^7^F_0_-^5^L_6_ transition. Moreover, several other weaker bands are assigned to transitions starting from the ground state ^7^F_0_ or thermally populated ^7^F_1_ level.

The emission spectra for the three TiO_2_/NaYF_4_: 10% Eu nanocomposite samples upon excitation at 532 nm, are shown in Fig. 6b. The spectra show bands due to emission decays from the ^5^D_1_ and ^5^D_0_ excited states of Eu^3+^ ions to the underlying ^7^F_J_ levels. The spectra are dominated by the emission bands due to the ^5^D_0_-^7^F_2_ hypersensitive transition, indicating that on average the Eu^3+^ ions are occupying sites with low symmetry. An objective has been used to simultaneously excite and collect the emission radiations. We found that the relative intensities of the signals measured for different positions on the thin film sample are different, denoting a slight inhomogeneity in thickness of the film on the entire 2 × 3 cm^2^ area. In the inset of Fig. 6b, an enlargement of the 574–582 nm spectral region is shown, highlighting the presence of bands due to the ^5^D_0_-^7^F_0_ transition of the Eu^3+^ ion. This band is due to a singlet-to-singlet transition and therefore it is very meaningful to investigate the number of different sites in which the lanthanide ions are placed. This band is present only for low symmetries Eu^3+^ lanthanide ion environments, and it indicates the occupancy of crystal sites with C_nv_, C_n_ or C_s_ symmetry^48^.The inset of Fig. 6b shows more than one peak for the samples, indicating that more than one site are present. Moreover, a difference in the ^5^D_0_-^7^F_0_ bands among the three samples is clearly observed, indicating that the irradiation impacts on the symmetry of the crystal sites occupied by the Eu^3+^ ions^49^.

Figure 6c shows the luminescence decay curves for the TiO_2_/β-NaYF_4_: 10% Eu^3+^composites samples after the different photocatalytic experiments, upon pulsed laser excitation at a wavelength of 532 nm. The emission decay curves (613 nm) have been fitted using a biexponential function (Eq. 2):

2 $$\mathrm{I}\left(\mathrm{t}\right)={\mathrm{A}}_{1}{\mathrm{e}}^{-\frac{\mathrm{t}}{{\uptau }_{1}}}+{\mathrm{A}}_{2}{\mathrm{e}}^{-\frac{\mathrm{t}}{{\uptau }_{2}}}$$

Where τ_i_ (i = 1, 2) represent the calculated decay lifetimes, while the A_i_ (i = 1, 2) are the corresponding amplitudes of the two decay components. The average lifetimes τ_av_ are calculated by Eq. 3:^50^

3 $${\uptau }_{\mathrm{av}}=\frac{\sum_{\mathrm{i}}{\mathrm{A}}_{\mathrm{i}}{\uptau }_{\mathrm{i}}^{2}}{\sum_{\mathrm{i}}{\mathrm{A}}_{\mathrm{i}}{\uptau }_{\mathrm{i}}}$$

The decay lifetimes and the amplitudes obtained by the fit, as well as the average lifetimes values, are reported in Table 3. From the obtained values, we can notice that the photocatalytic experiments affect the luminescence properties of the samples. In particular, the average lifetime decreases on passing from the as-prepared sample to the ones subjected to photocatalysis in a monotonic way, suggesting that the local environment of the lanthanide ions becomes less symmetric on increasing the number of photocatalytic cycles.

**Table 3:** Decay lifetimes τ_i_ (i = 1, 2), amplitudes A_i_(i = 1, 2), average lifetimes τ_av_ obtained for the TiO_2_/β-NaYF_4_: 10% Eu^3+^ nanocomposites.

	τ_1_ (ms)	A_1_	τ_2_ (ms)	A_2_	τ_av_ (ms)
As prepared	1.75 ± 0.04	0.35 ± 0.04	6.67 ± 0.02	0.60 ± 0.02	6.02 ± 0.03
After one photocatalytic cycle	2.13 ± 0.02	0.44 ± 0.03	6.96 ± 0.02	0.48 ± 0.02	5.91 ± 0.02
After three photocatalytic cycles	1.30 ± 0.01	0.58 ± 0.03	5.92 ± 0.01	0.30 ± 0.01	4.53 ± 0.01

This behavior suggests that the photocatalytic experiments have a relevant effect on the local environment of the lanthanide ions, in particular the ones located on the surface of the NaYF_4_ nanostructures, most probably more involved in the photocatalytic process. Moreover, the observed trend agrees with the photocatalytic recycling experiments, with the first cycle being the one in which the sample shows the major activity and the 2nd and 3rd ones showing a slightly smaller effect of the sample in the dye degradation. It is important to note that the average lifetime of the untreated sample is in good agreement with the values found in the literature for Eu^3+^ doped NaYF_4_ samples^51,52^.

## Conclusion

The present work provides a comprehensive study of the synthetic strategy, in-depth characterization and photocatalytic application tests of nanostructured composites TiO_2_ /β-NaYF_4_ suitably doped with luminescent lanthanide ions, which induce upconversion (Yb^3+^, Tm^3+^, Gd^3+^) or downshifting (Eu^3+^) properties. The synthesis of the pure NaYF_4_/TiO_2_ and the doped NaYF_4_/TiO_2_ composite films has been performed through a straightforward sol–gel/spin-coating method, which is a simple, industrially appealing and highly reproducible method, and selectively produces only the composite with the hexagonal NaYF_4_ phase, the best phase for energy conversion properties. The morphological characterization confirms the formation of porous and compact thin films over a large area for the different composite materials, suitable for photocatalysis application. Photoluminescence measurements have confirmed the upconverting properties of the NaYF_4_/TiO_2_ composite films due to the presence of the Yb^3+^, Tm^3+^, Gd^3+^ ions and the downshifting properties of the Eu^3+^ doped NaYF_4_/TiO_2_ composite film.

The photocatalytic activity of these nanostructured composites, tested for the degradation of the methylene blue, used as a model pollutant, reveals a different behavior depending on whether the irradiation occurs under visible or under UV light and as a function of the upconverting and downshifting properties of the composites.

The system showing the greatest degradation under UV irradiation is the downshifting NaYF_4_/TiO_2_: Eu^3+^ 10%. Indeed, this system has a higher photo-degradation ability in respect of the blank and, after being subjected to more irradiation cycles, it keeps a good efficiency in the photo-degradation of MB. The form of the photocatalyst is a pivotal aspect of the present study, since the photocatalytic sample is immobilized and not used as a dispersed phase. This is a crucial point for future industrial applications, since the active sample can be easily recovered and reused for successive cycles, as presently demonstrated.

This work represents a promising starting point for the production of nanostructured composites which can be used for water purification and can also be extended to other photocatalytic applications.

## Methods

### Materials

The P25 TiO_2_ nanoparticles with an average diameter of 20 nm were purchased from Aldrich and are composed of 80% anatase and 20% rutile. Methylene blue was purchased from Merck.

### Synthesis of the nanocomposites

The sol–gel/spin-coating process was applied to the synthesis of the blank β-NaYF_4/_TiO_2_ and the lanthanide-doped β-NaYF_4/_TiO_2_ samples. The synthesis of the undoped and lanthanide-doped NaYF_4_ was carried out in a water/ethanol solution of Na(hfa)·tetraglyme, Y(hfa)_3_·diglyme with the addition, in case of the lanthanide-doped phase, of the Ln(hfa)_3_·diglyme, with Ln = Yb^3+^, Gd^3+^, Tm^3+^ or Eu^3+^.

The Na(hfa)·tetraglyme, Y(hfa)_3_·diglyme and Ln(hfa)_3_·diglyme complexes were synthesized according to literature procedures^53–55^. For the sol hydrolysis, the trifluoroacetic acid (CF_3_COOH) was used as catalyst. The synthesis of the undoped NaYF_4_ sample (used as a blank) was carried out with the following molar ratio of precursors, solvent and catalyst:

1 mmol Y(hfa)_3_·diglyme: 1 mmol Na(hfa)·tetraglyme : 43 mmol C_2_H_5_OH : 1.5 mmol H_2_O : 0.4 mmol CF_3_COOH.

The synthesis of the lanthanide-doped NaYF_4_ sample was carried out with the following molar ratio of precursors, solvent and catalyst:

(1-x) mmol Y(hfa)_3_·diglyme: x mmol Ln(hfa)_3_·diglyme: 1 mmol Na(hfa)·tetraglyme : 43 mmol C_2_H_5_OH : 1.5 mmol H_2_O : 0.4 mmol CF_3_COOH.

The sol was aged at 60 °C under stirring for 20 h. Afterwards, 0.5 mmol of TiO_2_ nanoparticles have been added and suspended under stirring in the sol for 30 min, and spin-coated on Si (100) and glass substrates. The spin coating process was carried out using a Spin-Coater SPIN-150 SPS Europe, at 3000 rounds per minute (RPM), a time of 60 s and a speed rate of 1000 RPM/sec. The deposition process involves a multistep procedure in which spin-coating deposition is alternated to fast annealing steps at 400 °C in air for 10 min. After four steps, the films were annealed at 400 °C in air for 1 h. The composite samples for the photocatalytic applications were spun on 2 cm × 3 cm glass substrate.

### Characterization of the nanocomposites

The film structure was analysed by X-ray diffraction (XRD) in grazing incidence mode (0.5°) using a Smartlab Rigaku diffractometer, equipped with a rotating anode of Cu Kα radiation operating at 45 kV and 200 mA. The film surface morphology was investigated using the field emission scanning electron microscope (FE-SEM), ZEISS Supra 55 VP. The atomic composition of the sample was determined by performing energy dispersive X-ray (EDX) analysis, using an INCA-Oxford windowless detector, having a resolution of 127 eV as the FWHM of Mn Kα.

### Nanocomposites luminescence characterization

The conversion emission spectra for the Yb^3+^, Gd^3+^, Tm^3+^ doped samples and Eu^3+^ doped samples, were measured, respectively, using a 980 nm diode laser (CNI Optoelectronics Tech) and a 532 nm diode laser as excitation sources, Czerny-Turner monochromator (Andor, Shamrock 500i) equipped with a 1200 lines/mm grating and an iDus CCD camera cooled at -80 °C. The emission signal was collected with a 40 × microscopy objective (Nikon, Plan Fluor) using, respectively, a dichroic mirror (Semrock, 925 nm edge BrightLine beam splitter) and a 70:30 beam splitter. The decay curves were acquired with a R928 photomultiplier (Hamamatsu) and a 500 MHz Oscilloscope (LeCroy, Waverunner), using a signal generator connected to a 532 nm diode laser. Excitation spectra for the Eu^3+^ doped samples were measured using a Fluorolog-3 (Horiba-Jobin Yvon), spectrophotometer, with an optical spectral resolution of 0.5 nm.

### Photocatalytic activity

The study of the photocatalytic activity of the nanostructured TiO_2_/Ln-doped fluoride composites towards MB was carried out by irradiating the samples from the top with visible light or UV light. The used visible light source was an artificial lamp (Ingenieurbüro Mencke & Tegtmeyer GmbH, Germany) controlled by a Susicontrol software (version 2.9.0) for the monitoring of the light intensity performed by means of a silicon irradiance sensor. As reported by the producer, the wavelength ranges from 400 to 1100 nm. The beaker was placed at 15 cm from the light source (distance between the bottom of the beaker and the irradiating source) and the visible light irradiance was of 98 W/m^2^. The samples were suspended through Au wires in MB aqueous solution (Fig. S10). For the experiments under UV irradiation, an UV lamp (UMEX GmbH), equipped with six Philips 8 W mercury florescent tubes (E_max_ at 365 nm) was used. The distance between the base of the beaker and the irradiating source was 15 cm. The light irradiance was measured by an UV34 radiation Meter (PCE) and it was found to be around 18 W/m^2^. For both the experiments under UV or visible light, a 2 cm × 3 cm sample was fixed on a gold support inside a beaker and immersed in 50 ml of MB aqueous solution (1.8 × 10^−5^ M) under radiation (visible or UV) and constant stirring. The ultrapure water used was produced by a MembraPure Astacus system (MembraPure GmbH, Hennigsdorf, Germany) and its conductivity was 0.055 µS/cm at T = 30 °C. Prior to the degradation experiments, the MB solution and the substrate inside the beaker, covered by aluminum, were firstly subjected to a dark adsorption process under constant stirring for 30 min to achieve an adsorption–desorption equilibrium of the dye on the surface of the nanocomposite. To analyze the degradation of MB by means of the nanostructured TiO_2_/Ln-doped fluoride composites, measurements were taken at defined time intervals from 0 to 240 min. The photo-degradation of MB was monitored by analyzing the decrease of absorbance at the MB maximum absorption peak (663 nm) using a Varian CARY-100 UV–vis spectrophotometer. A control (bare solution of MB) to evaluate the photolysis of MB and one with a sample of NaYF_4_/TiO_2_ without dopants were also tested under the same irradiation and experimental conditions.

For the recycling experiments under UV irradiation, the samples were washed with water and then reused for the photocatalytic degradation of a fresh solution of MB (1.8 × 10^−5^ M). This procedure was repeated 3 times.

## Supplementary Information

Supplementary Information.
