# Supplementary material for: Synthesis, characterization and photocatalytic properties of nanostructured lanthanide doped β-NaYF4/TiO2 composite films
Source: Sci Rep. 2022 Aug 12;12:13748. doi: 10.1038/s41598-022-17256-2 (PMC9374679; doi:10.1038/s41598-022-17256-2)
Supplement: Supplementary file 1 — Supplementary Information. [file 41598_2022_17256_MOESM1_ESM.pdf]

Supporting information

Synthesis, characterization and photocatalytic properties of nanostructured lanthanide doped β-$NaYF_4$/TiO_2 composite films

Fabiana M. Pennisi,[a,†,∫] Anna L. Pellegrino,[a,†] Nadia Licciardello,[b,‡,*] Claudia Mezzalira,[c] Massimo Sgarzi,[b,#] Adolfo Speghini,[c] Graziella Malandrino,[a,*] and Gianaurelio Cuniberti[b,*]

[a] *Dipartimento di Scienze Chimiche, Università di Catania, and INSTM UdR Catania, Viale A. Doria 6, I-95125 Catania, Italy.*

[b] *Institute for Materials Science, Max Bergmann Centre of Biomaterials and Dresden Center for Nanoanalysis, TU Dresden, 01062, Dresden, Germany.*

[c] *Nanomaterials Research Group, Dipartimento di Biotecnologie, Università di Verona and INSTM, UdR Verona, Strada Le Grazie 15, I-37134 Verona, Italy.*

[†] These authors have contributed equally to the manuscript.
[∫] Current address: ST Microelectronics, Strada Primosole 50, 95121, Catania, Italy.
[‡] Current address: Department of Drug and Health Sciences, University of Catania, Viale Andrea Doria 6, I-95125 Catania, Italy
[#] Current address: Department of Molecular Sciences and Nanosystems, Ca' Foscari University of Venice, Via Torino 155, 30170 Venezia Mestre, Italy

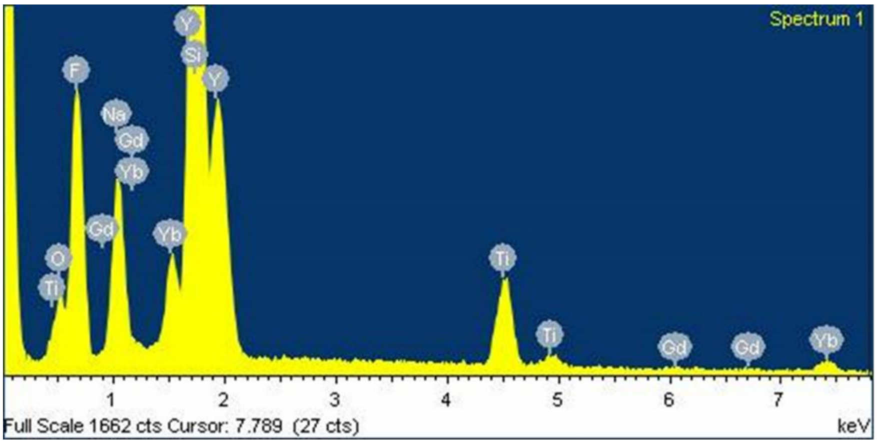

Element	Atomic%
O (K)	16.20
F (K)	34.45
Na (K)	8.81
Si (K)	27.03
Ti (K)	5.12
Y (L)	6.67
Gd (L)	0.20
Yb (L)	1.52

Fig. S1. EDX spectrum of the TiO_2/β-$NaYF_4$:Yb^{3+},Gd^{3+},Tm^{3+} nanocomposite.

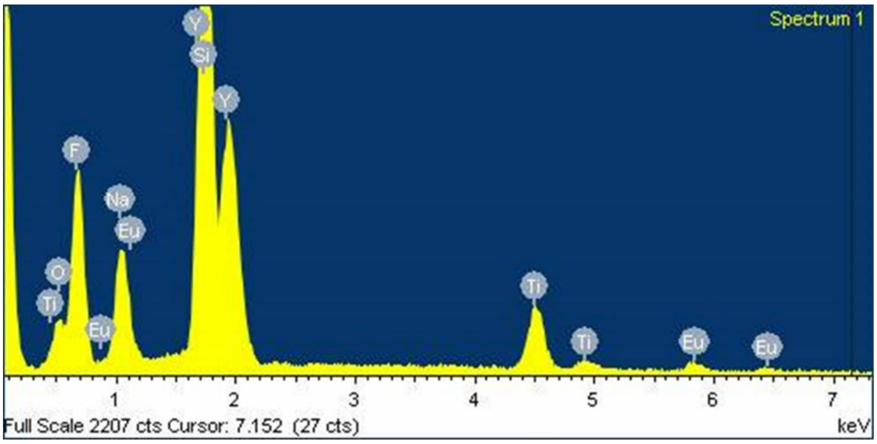

Element	Atomic%
O (K)	17.99
F (K)	36.43
Na (K)	8.87
Si (K)	22.14
Ti (K)	5.08
Y (L)	8.46
Eu (L)	1.02

Fig. S2. EDX spectrum of the TiO_2/β-$NaYF_4$: Eu^{3+} nanocomposite.

Site 3 Eu (green),Y (red), Na (orange), F(blue), Ti (white)

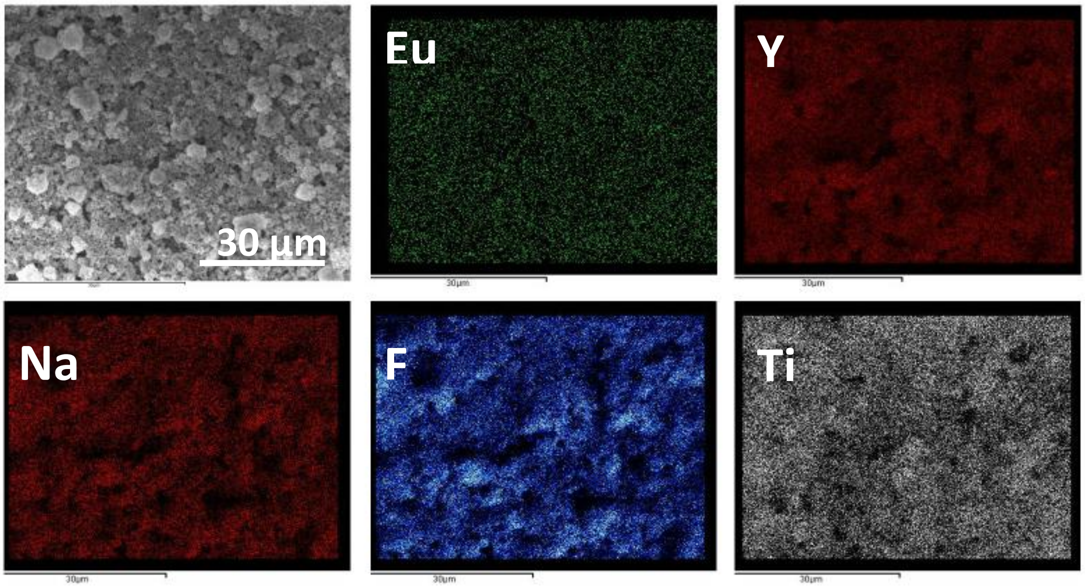

Fig. S3. EDX elemental mapping for the TiO_2/β-$NaYF_4$: Eu^{3+} nanocomposite.

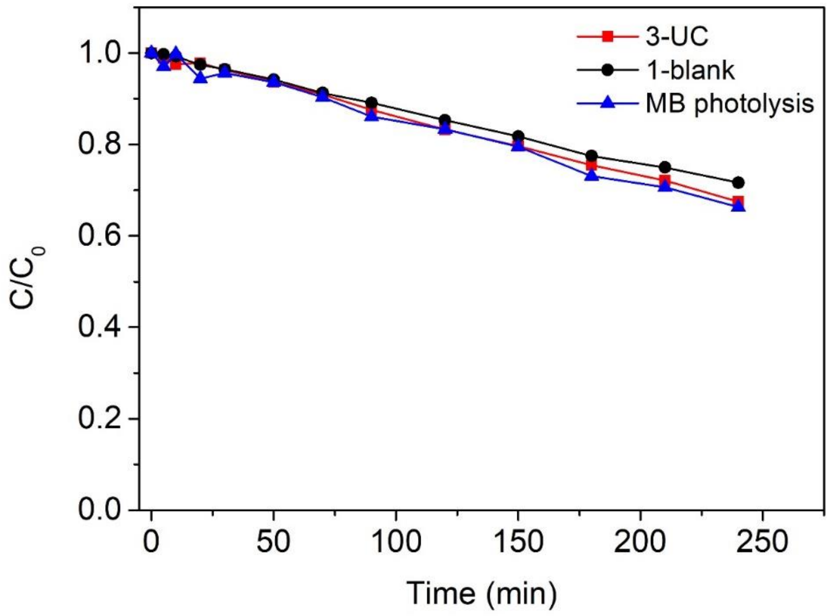

Fig. S4 Variation of concentration of an aqueous MB solution (1.8 × 10^{-5} M) versus time under only visible light irradiation (photolysis in blue), in the presence of the sample 1-blank and visible light irradiation (in black) and in the presence of sample 3-UC and visible light irradiation (in red).

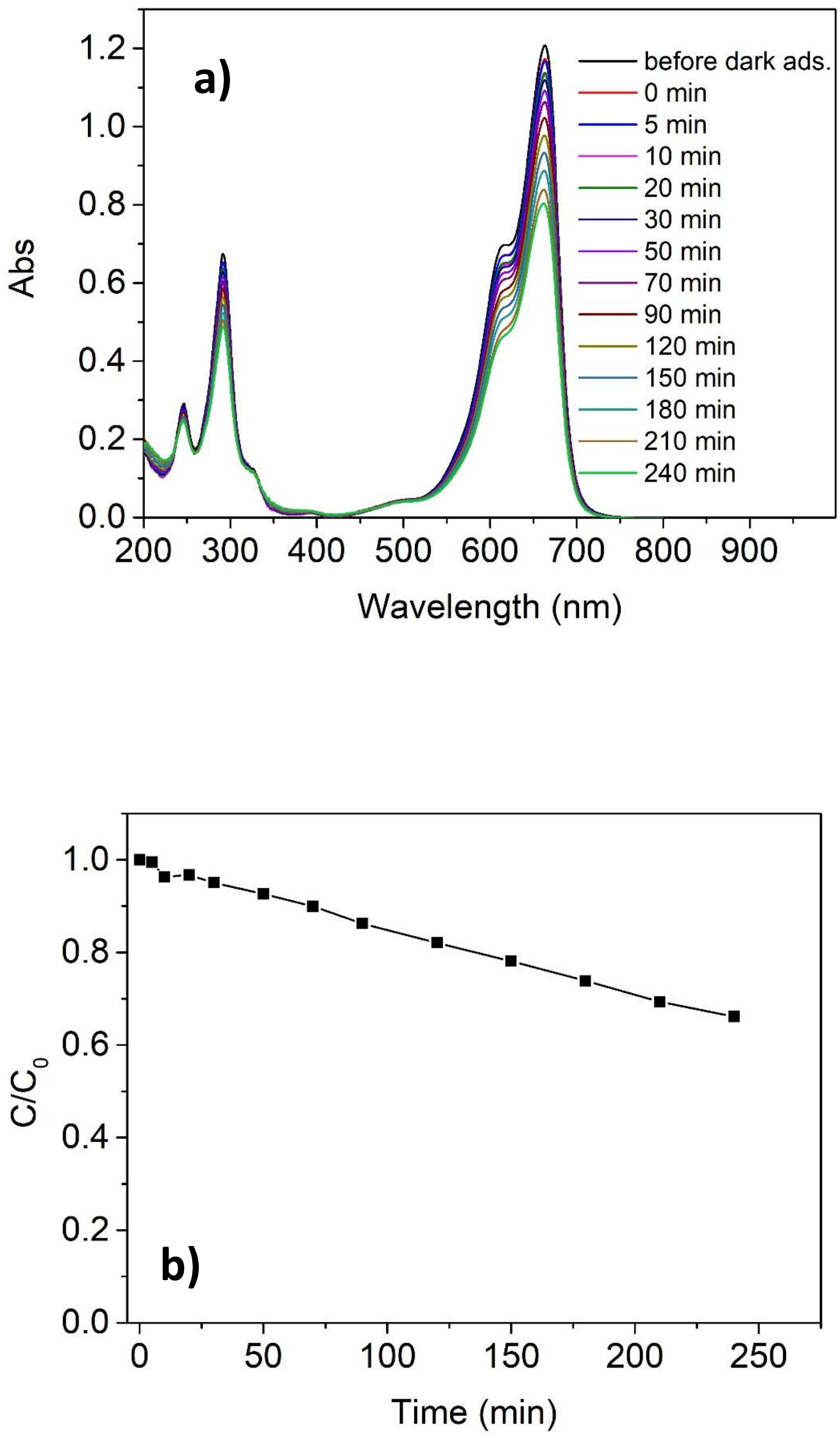

Fig. S5 Photodegradation of an aqueous MB solution (1.8×10^{-5} M) in the presence of sample 2-UC: TiO_2/$NaYF_4$: Yb (20%), Gd^{3+}(2%), Tm^{3+}(1%) under visible light: (a) variation of the UV-Vis absorption spectra of the MB solution in time during the photodegradation (the first spectrum represents the spectrum acquired before the 30 minutes of dark adsorption and the zero time is exactly at 30 minutes after dark adsorption); (b) Variation of the concentration of MB in time during the photodegradation.

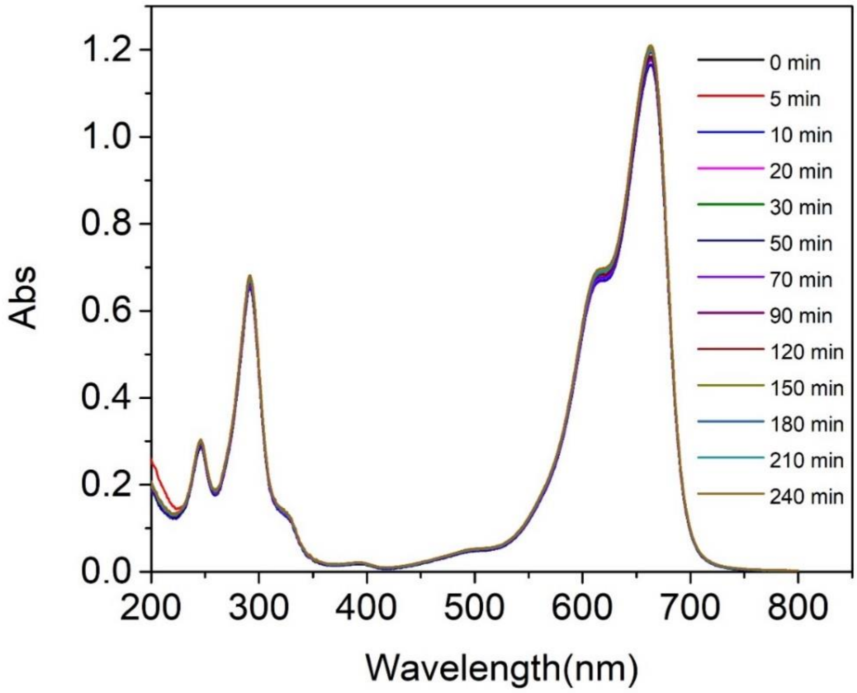

Fig. S6. Photolysis of an aqueous MB solution (1.8 × 10^{-5} M) under UV light irradiation

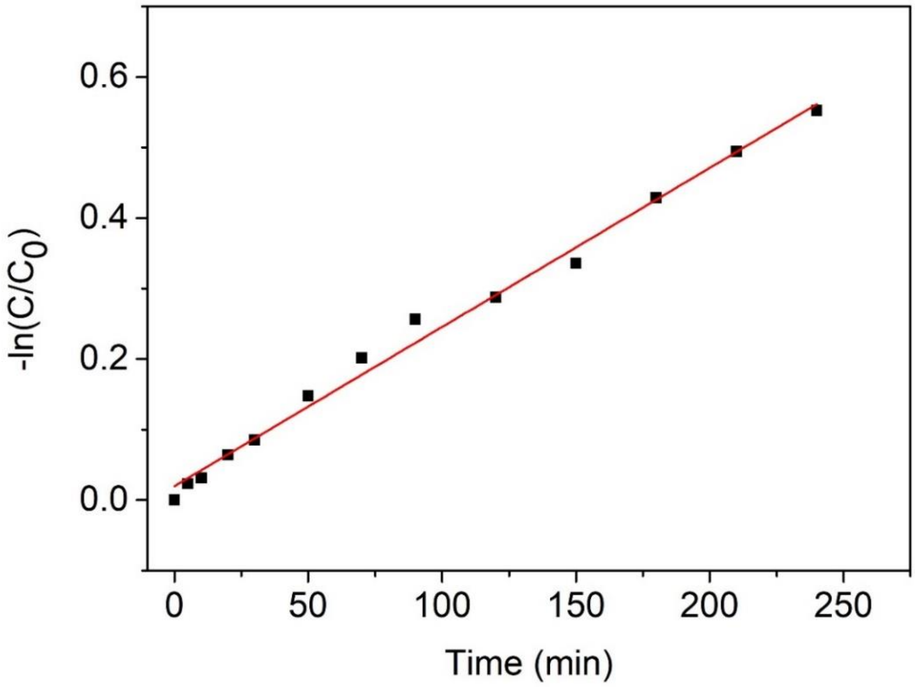

Fig. S7: Linear relationship of −ln(C_t/C_0) vs. time for 4-DS.

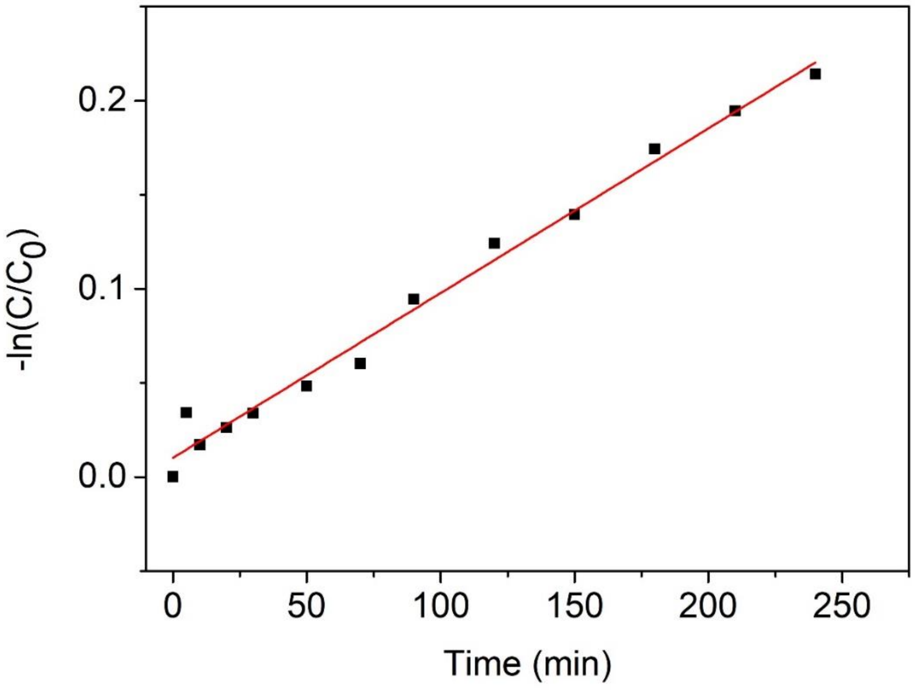

Fig. S8 Linear relationship of −ln(C_t/C_0) vs. time for 1-blank.

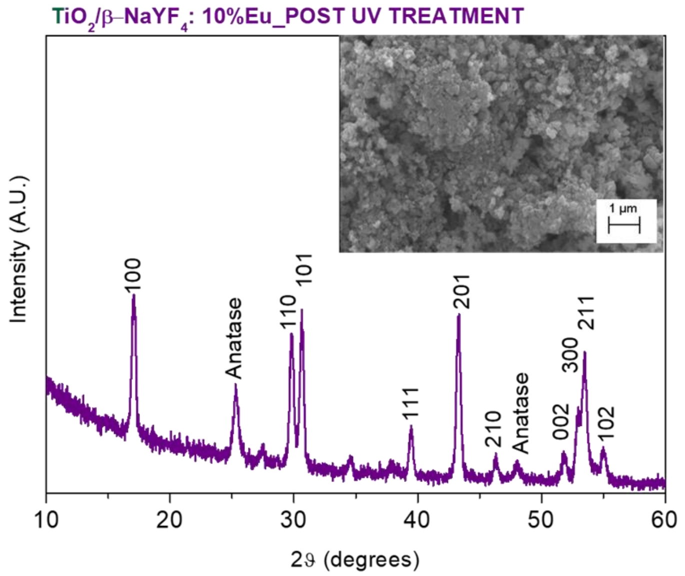

Fig. S9 XRD pattern and FE-SEM image of the 4-DS sample (TiO_2/β-$NaYF_4$: 10%Eu) after 3-recycling tests.

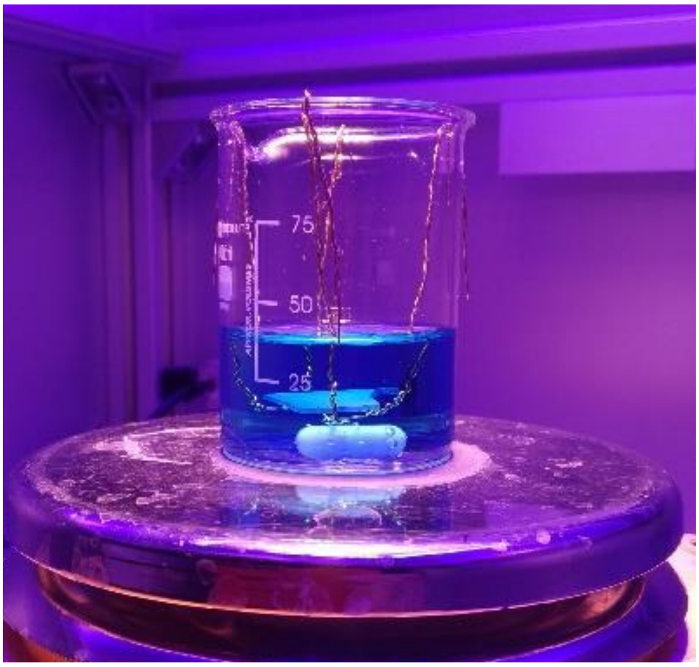

Fig. S10. Set-up for the photocatalytic experiments, showing the sample suspended through Au wires into the aqueous MB solution.
